# A Prospective Assessment of the Diagnostic Value of Contrast-Enhanced Ultrasound, Dynamic Computed Tomography and Magnetic Resonance Imaging for Patients with Small Liver Tumors

**DOI:** 10.3390/jcm8091353

**Published:** 2019-09-01

**Authors:** Chih-Yang Hsiao, Po-Da Chen, Kai-Wen Huang

**Affiliations:** 1 Graduate Institute of Clinical Medicine, National Taiwan University College of Medicine, Taipei 10048, Taiwan; 2 Department of Surgery, National Taiwan University Hospital, Taipei 10002, Taiwan; 3 Department of Traumatology, National Taiwan University Hospital, Taipei 10002, Taiwan; 4 Hepatitis Research Center, National Taiwan University Hospital, Taipei 10048, Taiwan

**Keywords:** contrast-enhanced ultrasound, computed tomography, diagnosis, hepatocellular carcinoma, liver tumor, magnetic resonance imaging

## Abstract

Aim: This prospective study assessed the diagnostic value of contrast-enhanced ultrasound (CEUS) using long Kupffer phase enhancement for adults with liver tumor size of less than 3 cm. Performance comparisons were also conducted with dynamic computed tomography (CT) and magnetic resonance imaging (MRI). Background: CEUS has emerged as a reliable image modality, since the development of second-generation contrast medium with long Kupffer phase enhancement. Nonetheless, dynamic CT and MRI are currently the standard imaging tools for the diagnosis of liver cancers, and the diagnostic value of CEUS for liver cancer has yet to be universally accepted. Methods: Sixty-six adult patients suspected of having liver tumors smaller than 3 cm underwent CEUS, dynamic CT, and MRI examinations independently. Subsequent tumor biopsies were used to verify the diagnostic performance of the three imaging modalities. Results: The diagnostic odds ratio (DOR, 95% CI) for hepatocellular carcinoma were as follows: CEUS (52.8, 11.4–243), MRI (29.29, 7.36–116), and CT (19.43, 5.44–69.4); for metastasis: CEUS (200, 19.1–2095), MRI (24, 5.05–114), and CT (32, 6.56–156); and all liver malignancy: CEUS (260, 12.7–5310), MRI (2.57, 0.55–12.1), and CT (5.22, 1.25–21.8). CEUS achieved the best differentiation performance. Conclusions: CEUS outperformed dynamic CT and MRI in terms of diagnostic performance when dealing with small liver tumors (<3 cm).

## 1. Introduction

Conventional ultrasound, dynamic computed tomography (CT), and magnetic resonance imaging (MRI) are the most prevalent imaging methods used for the characterization of liver lesions. Conventional ultrasound is convenient and cost-effective as a first-line screening test; however, the lack of dynamic imaging to profile vascularity means that it is unable to match the accuracy of dynamic CT and MRI [1]. Second-generation contrast agents phagocytosed by Kupffer cells (i.e., Kupffer imaging) [2] and contrast-enhanced ultrasound (CEUS) have proven highly reliable as diagnostic tools for liver lesions as small as 1 cm [3,4,5]. These methods facilitate the characterization of liver lesions through the peripheral intravenous injection of contrast medium, comprising gaseous microbubbles stabilized within a shell [6].

CEUS has a number of advantages over dynamic CT and MRI (Table 1). It is more convenient, it can be administered in less time, and it does not involve ionizing radiation. The fact that the contrast medium used in CEUS is metabolized by the respiratory system rather than through renal excretion means that it is suitable for patients with renal impairment. Although CEUS is widely recommended for focal liver lesions [7], its diagnostic value (particularly for liver malignancy) has not been clearly established. Hepatocellular carcinoma (HCC) is the most prevalent primary liver malignancy and a leading cause of cancer-related death worldwide [8,9]. The highly specific vascular presentation and imaging patterns of HCC mean that under most conditions, a reliable diagnosis can be based solely on imaging studies; i.e., without tissue evidence. Current consensus guidelines for the diagnosis of HCC are based primarily on tri-phasic CT or MRI [7,10,11]. A number of studies have addressed the diagnostic value of CEUS in HCC [12,13,14,15] and its usefulness in differentiating malignancy from benign lesions [16,17,18]. However, few of the above-mentioned studies used newly developed contrast media, and most of that research was limited by small sample sizes and/or a retrospective study design. Our objective in this prospective study, was to assess the diagnostic value of CEUS using the second-generation contrast medium Sonazoid^TM^ (GE Healthcare, Oslo, Norway), as a single diagnostic modality. We then compared the efficacy of this scheme with that of CT and MRI, in diagnosing liver tumors smaller than 3 cm.

## 2. Experimental Section

### 2.1. Trial Design and Participants

This study began on 1 December, 2017 and finished on 1 December, 2018. The first participant was recruited on 14 December, 2017 and the last one was recruited on 26 November, 2018. Patients were recruited from outpatient clinics. Inclusion criteria included the detection of solitary liver tumors smaller than 3 cm, which were suspected of malignancy (i.e., excluding simple cyst and hemangioma) using conventional ultrasound. Informed consent was obtained from the patients prior to enrollment. Using power = 80%, with one-sided alpha = 0.025, the sample size was estimated in accordance with the specificity and sensitivity reported in the literature. Assuming that the sensitivity of CT or MRI is 0.92 and the proportion of discordance between CEUS and CT is 0.14, then a sample size of 60 should be sufficient for a non-inferiority test with a margin of 0.15. This study was approved by the Research Ethics Committee of National Taiwan University Hospital (NTUH REC: 201706079MIPA), and the study was carried out in accordance with the approved guidelines (ClinicalTrials.gov number, NCT03267290).

### 2.2. Examinations and Procedures

The three image modalities (dynamic CT, dynamic MRI, and CEUS) were implemented in accordance with standard protocols. Dynamic CT and MRI were quadruple-phase imaging series (pre-contrast phase, arterial phase, portal venous phase, and equilibrium phase), in which non-ionic iodine-based contrast medium was used for CT, and gadolinium-based medium was used for MRI. During CT or MRI exams, the dosage of the contrast medium was based on the body weight of the patient. The contrast medium was injected using an automated contrast injector to reduce inter-operator difference, which might otherwise affect the quality of the resulting scans. CEUS was performed by a physician familiar with abdominal ultrasonography. During CEUS examination, the patient stayed in a supine position. Ultrasound was performed first in B-mode to identify the suspicious lesion, and then switched to contrast mode prior to the injection of the contrast medium. Sonazoid^TM^ (GE Healthcare, Oslo, Norway), which consists of perfluorobutane microbubble, was injected (0.01 mL/kg) intravenously (manually) as a contrast medium. After injection of the contrast medium, imaging was monitored during the arterial phase (20–40 seconds post-injection) and Kupffer phase (10 min post-injection). Diagnosis was made based on this dynamic imaging of enhancing patterns according to a time sequence or phase. Following the completion of the three imaging examinations, tumor biopsies were performed under image guidance to determine the histopathology of the tumor. Histopathological analysis was performed by a pathologist using the standard method in accordance with the guidelines of the Pathology Department in our hospital.

To evaluate the safety of CEUS we recorded the vital signs (blood pressure, heart rate, and body temperature) throughout the exam and conducted laboratory examinations (hematologic, renal, and hepatic function test) one day after the exam. Any associated side effects or adverse events were recorded.

### 2.3. Data Interpretation

Analysis of the imaging and histopathological results was performed independently by four different physicians. The interpretation of CT and MRI scans was performed by two different radiologists. The interpretation of CEUS results was performed by the physician who performed the CEUS examination. The histopathology was reported by a pathologist. Physicians involved in this study were blinded from the results of other examinations while formulating their interpretations. To fit the goal of this study, the interpretation of all imaging data was reported in a structured manner to ensure a precise clinical diagnosis, using the following classification scheme: 0—not detected, 1—HCC, 2—metastasis, 3—benign tumor, 4—uncertainty. The pathology results were treated as a reference diagnosis, which was originally reported by textual description and later classified using the following schema: 0—no tumor, 1—HCC, 2—metastasis, 3—benign tumor.

### 2.4. Outcomes and Statistical Analysis

The outcome of interest in this study was the diagnostic value of using CEUS as a tool to characterize liver tumors smaller than 3 cm. We also compared CEUS, CT, and MRI in terms of sensitivity, specificity, positive predictive value (PPV), and negative predictive value (NPV). The diagnostic odds ratio (DOR), (Sensitivity/(100 − Specificity))/((100 − Sensitivity)/Specificity), was calculated as a single indicator of diagnostic performance [19]. To evaluate the role of CEUS in real clinical practice, we calculated the specificity of combining two imaging exams. The receiver operating characteristic (ROC) curve and area under the curve (AUC) were also derived. Data are presented as mean +/− standard deviation, or a number with percentage, as appropriate. Statistical analysis was performed using Excel 2010 (Microsoft Corporation, Redmond, WA, USA) or SPSS 18 (IBM, Chicago, IL, USA) for Windows.

## 3. Results

### 3.1. Patient Characteristics and Safety

During the study period, a total of 66 patients were enrolled in the study (Table 2). Twenty-one (21/66; 31.8%) participants were women. The participants had a mean age of 63.3 years, and a mean body-mass index (BMI) of 22.8. Thirty-three (33/66; 50%) patients had a normal liver background, and others had cirrhosis (26/66; 39.4%) and fatty liver (7/66; 10.6%). The mean tumor diameter was 1.6 cm. All patients underwent analysis using all three imaging systems as well as a pathological examination of the tumor. The most frequent pathological diagnosis was HCC (41/66; 62.1%), followed by metastatic tumor (15/66; 22.7%), and benign tumor (6/66; 9.1%). No patient underwent any adverse event at any point in the study.

### 3.2. Diagnostic Performance of CEUS, MRI, and CT

Table 3 lists the sensitivity, specificity, and DOR of the three imaging modalities in this study. In the diagnosis of HCC, MRI had the best sensitivity (37/41; 90.2%), followed by CEUS (36/41; 87.8%), whereas CEUS had the best specificity (22/25; 88%), followed by CT (20/25; 80%). As for diagnosis of metastatic tumors, CEUS had the best sensitivity (12/15; 80%) and specificity (50/51; 98%), followed by CT (with sensitivity of 66.7% (10/15) and specificity of 94.1% (48/51)) and MRI (with sensitivity of 60% (9/15) and specificity of 94.1% (48/51)). Overall, for the diagnosis of all malignancies (HCC and metastatic tumor with the same diagnosis of “malignancy”), CEUS still had the best sensitivity (52/56; 92.9%) and specificity (10/10; 100%), followed by CT (with sensitivity of 83.9% (47/56) and specificity of 50% (5/10)) and MRI (with sensitivity of 85.7% (48/56) and specificity of 30% (3/10).

Table 3 lists the positive predictive value (PPV) and negative predictive value (NPV) of the three imaging modalities. For HCC, CEUS had the best PPV (92.3%), followed by CT (87.2%), and MRI (86%). MRI had the best NPV (82.6%), followed by CEUS (81.5%), and CT (74.1%). As for all-malignancy (combined HCC and metastatic tumor as a single diagnosis), CEUS had the best PPV and NPV.

When using DOR as a single indicator of test performance, CEUS had the best diagnostic performance in HCC, metastatic tumor, or all-malignancy diagnosis (Table 3).

### 3.3. Diagnostic Specificity When Combining Two Imaging Modalities

Table 4 lists the diagnostic specificity of two of the imaging modalities used in combination. In the diagnosis of HCC, the specificity was as follows: CEUS + CT (24/25; 96%), CEUS + MRI (23/25; 92%), and MRI + CT (23/25; 92%). In the diagnosis of liver metastasis, the specificity was as follows: CEUS + CT (51/51; 100%), CEUS + MRI (51/51; 100%), and MRI + CT (50/51; 98%). In all-malignancy diagnosis, the specificity was as follows: CEUS + CT (10/10; 100%), CEUS + MRI (10/10; 100%), and MRI + CT (9/10; 90%).

### 3.4. ROC Curve and AUC

**Figure 1:**
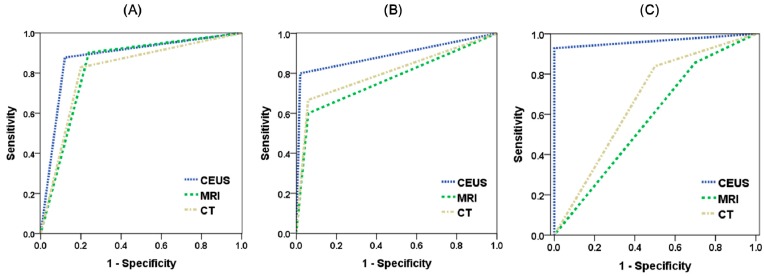
The receiver operating characteristic (ROC) curve and area under the curve (AUC) of the three imaging modalities: (**A**), for diagnosis of HCC; (**B**), for diagnosis of liver metastasis; (**C**), for diagnosis of all-malignancy.

Figure 1 presents the ROC curves and AUC of the three image modalities. In the diagnosis of HCC (Figure 1A), all three imaging modalities presented good discriminative power as indicated by the AUC:CEUS (0.879), MRI (0.831), and CT (0.815). In the diagnosis of a metastatic tumor (Figure 1B), CEUS and CT presented good discriminative power, as indicated by the AUC:CEUS (0.890) and CT (0.804), whereas MRI presented fair discriminative power of 0.771. As shown in Figure 1C, in all-malignancy diagnosis (combining HCC and metastatic tumor as a single diagnosis), CEUS had excellent discriminative power (AUC = 0.964); however, MRI had no discriminative power (AUC of 0.579) and CT had poor discriminative power (0.670).

## 4. Discussion

Our results illustrated the diagnostic value of CEUS (using Sonazoid^TM^) for patients suspected of having a liver tumor smaller than 3 cm. CEUS outperformed dynamic CT and MRI in discriminating among liver malignancies.

**Figure 2:**
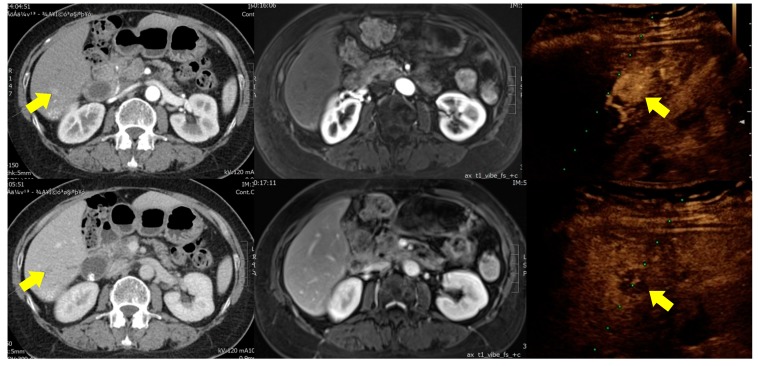
A 65-year-old woman with chronic hepatitis B infection was shown to have an S6 liver tumor. CT scans revealed trace enhancement in the arterial phase (left upper part) and mild wash-out in the portal phase (left lower part). MRI exam failed to detect the tumor (middle part). CEUS analysis showed strong enhancement in the arterial phase (right upper part) and typical wash-out in the Kupffer phase (right lower part). Biopsy confirmed a diagnosis of hepatocellular carcinoma.

Focal liver lesions are easily identified using conventional B-mode ultrasound; however, the primary concern in clinical practice is whether they are malignant. Diagnosing lesions based solely on imaging results is inherently limited by the heterogenic presentation of tumors. Most clinical guidelines permit a diagnosis of HCC without histopathological evidence; however, atypical image presentations are not uncommon (Figure 2). Furthermore, differences in pathology manifest as differences in imaging results. Thus, conclusive clinical diagnosis requires that tumor vascularity be visualized using dynamic images. In the event that a suspicious liver lesion is encountered, dynamic CT and MRI are currently the standard imaging modalities used for differential diagnosis. Nonetheless, CT and MRI have a number of inherent limitations. Obtaining a CT scan of the abdomen (with and without contrast medium) exposes a person to approximately 20 millisieverts, which is equal to about seven years of average background radiation exposure [20]. MRI is a safer exam in terms of radiation exposure; however, it is much more expensive and time-consuming (approximately 40 min per session). Furthermore, the strong magnetic field prevents its use for patients with metallic objects, such as an implanted defibrillator or pacemaker, clips for brain aneurysms, and metal stents. Furthermore, the nephrotoxicity of the contrast medium used in CT and MRI limits its use in patients with chronic kidney disease.

CEUS provides the advantages of conventional ultrasound (no radiation, rapid examinations, and portability) as well as the advantages of CT and MRI (clearer characteristic features and vasculature of a focal lesion). The lack of nephrotoxicity, radiation, and a strong magnetic field means that CEUS is applicable to a wider range of patients. Note also that the cost of a single CEUS examination is on par with that of dynamic CT scans. Many patients facing an elevated risk of liver malignancy (e.g., those with chronic hepatitis), or a history of cancer (e.g., hepatocellular carcinoma or colorectal cancer liver metastasis), have to undergo repeated dynamic imaging exams during follow-up. In such cases, CT scans would impose risks pertaining to accumulated radiation exposure, whereas MRI exams would impose a heavy economic burden.

It is essential to obtain a definite diagnosis (including tumor stage) prior to initiating treatment. At present, clinicians routinely use serial CT plus MRI to enhance diagnostic specificity, thanks to their effectiveness in detecting the local invasion of adjacent organs as well as distant metastasis, which are essential for tumor staging. Under these circumstances, it appears that using CEUS in combination with CT or MRI might be an ideal solution. In this study, such combinations were shown to achieve specificity comparable or even better than that afforded by MRI in conjunction with CT. In recent years, medical associations have increasingly recommended CEUS as a diagnostic tool for focal liver lesions in their clinical practice guidelines. Our results support the use of CEUS with Sonazoid^TM^ as a reliable imaging modality for the diagnosis of liver tumor. Our results also indicate that CEUS could be used as a standard diagnostic tool for monitoring tumor recurrence after treatment, due to the fact that it outperforms CT and MRI in terms of diagnostic performance.

This study is subject to a number of limitations. First, all of the procedures were performed by only one physician familiar with CEUS techniques; therefore, interrater reliability could not be confirmed. Second, the study was conducted on a relatively small number of participants. Nonetheless, this study has other strengths. This was a prospective study in which the diagnostic performance of CEUS was compared directly with that of dynamic CT and MRI. The study was also conducted in a tertiary care facility with team members specializing in the use of these imaging modalities, such that the quality of the resulting images and diagnosis accuracy were both high. Furthermore, every patient underwent tumor biopsy to provide a reference by which to verify the diagnostic results. Our results demonstrated the diagnostic value of CEUS in patients with suspicious solitary liver tumors <3 cm, with diagnostic performance superior to that of CT and MRI. These results are a clear indication of its potentials as a standard diagnostic tool for the characterization of liver lesions.

## 5. Conclusions

Our study results indicate that the diagnostic performance of CEUS is superior to that of dynamic CT and MRI when dealing with small liver tumors (<3 cm). Using CEUS in combination with CT or MRI could enhance diagnostic specificity for liver malignancy and tumor staging. Furthermore, CEUS could be used as a standard diagnostic tool for monitoring liver tumor recurrence after treatment.

## Figures and Tables

**Table 1:** Comparison between contrast-enhanced ultrasound (CEUS), dynamic computed tomography (CT), and magnetic resonance imaging (MRI).

	CEUS	Dynamic CT	MRI
Ionizing radiation	No	Yes	No
Nephrotoxicity	No	Yes	Yes
Time needed	15 min	10 min	40 min
Technical need	Yes	No	No
Cost	260 USD	220 USD	500 USD

**Table 2:** Baseline characteristics of participants in this study.

Characteristic	Overall Participants (*n* = 66)
Gender (male:female)	45:21
Age (year)	63.3 ± 9.3
Body-mass index	22.8 ± 2.4
Liver characteristics	
normal	33 (50.0)
cirrhosis	26 (39.4)
fatty liver	7 (10.6)
Tumor size (cm)	1.6 ± 0.7
<1 cm	16 (24.2)
1–2 cm	27 (40.9)
2–3 cm	23 (34.8)
Pathology result	
no tumor	4 (6.1)
hepatocellular carcinoma	41 (62.1)
metastatic tumor	15 (22.7)
benign tumor	6 (9.1)

Data are presented as number (percentage) or mean ± standard deviation.

**Table 3:** Performance of CEUS, MRI, and CT in the diagnosis of liver tumors smaller than 3 cm.

	HCC					Liver Metastasis					All Malignancy				
	Sen	Spe	DOR (95% CI)	PPV	NPV	Sen	Spe	DOR (95% CI)	PPV	NPV	Sen	Spe	DOR (95% CI)	PPV	NPV
CEUS	87.8	88	52.8 (11.4–243)	92.3	81.5	80	98	200 (19.1–2095)	92.3	94.3	92.9	100	260 (12.7–5310)	100	71.4
MRI	90.2	76	29.29 (7.36–116)	86	82.6	60	94.1	24 (5.05–114)	75	88.9	85.7	30	2.57 (0.55–12.1)	87.3	27.3
CT	82.9	80	19.43 (5.44–69.4)	87.2	74.1	66.7	94.1	32 (6.56–156)	76.9	90.6	83.9	50	5.22 (1.25–21.8)	90.4	35.7

CEUS, contrast-enhanced ultrasound; CI, confidence interval; CT, computed tomography; DOR, diagnostic odds ratio; HCC, hepatocellular carcinoma; MRI, magnetic resonance imaging; NPV, negative predictive value; PPV, positive predictive value; Sen, sensitivity; Spe, specificity.

**Table 4:** Diagnostic specificity of combinations of CEUS, MRI, and CT for the diagnosis of liver tumors smaller than 3 cm.

Combination *	HCC	Liver Metastasis	All Malignancy
	Specificity	Specificity	Specificity
CEUS + CT	96	100	100
CEUS + MRI	92	100	100
MRI + CT	92	98	90

* Combination indicates that the diagnosis was based on double positive results from the two imaging exams. CEUS, contrast-enhanced ultrasound; CT, computed tomography; HCC, hepatocellular carcinoma; MRI, magnetic resonance imaging.
